# Central venous access device adverse events in pediatric patients with cancer: a systematic review and meta-analysis

**DOI:** 10.1007/s00520-024-08853-0

**Published:** 2024-09-16

**Authors:** Jenna L. Nunn, Mari D. Takashima, Erin M. Wray-Jones, Trisha A. Soosay Raj, Diane M. T. Hanna, Amanda J. Ullman

**Affiliations:** 1 Children’s Health Queensland Hospital & Health Service, Brisbane, Australia; 2 The University of Queensland, Brisbane, Australia; 3 Griffith University, Gold Coast, Australia; 4 Sunshine Coast University Hospital, Sunshine Coast, Australia; 5 The University of Melbourne, Melbourne, Australia; 6 Murdoch Children’s Research Institute, Melbourne, Australia; 7 The Walter &, Eliza Hall Institute, Melbourne, Australia; 8 The Royal Children’s Hospital, Melbourne, Australia

**Keywords:** CVAD, Central catheters, Pediatrics, Complications, Oncology

## Abstract

**Purpose:**

To systematically review the proportion and incidence of CVAD-associated complications in pediatric patients with cancer.

**Methods:**

PubMed, Embase, and the Cumulative Index of Nursing and Allied Health Literature were searched from 2012 to 2022. Cohort studies and the control arm of randomized controlled trials, which reported CVAD-associated complications in pediatric patients aged 0–18 years, were included. CVAD complications were defined as CVAD failure, central line–associated bloodstream infection (CLABSI), local infection, occlusion, CVAD-associated venous thromboembolism, dislodgement/migration, breakage/rupture, and dehiscence. The pooled proportion and incidence rate (IR) for each CVAD-associated complication were reported.

**Results:**

Of 40 included studies, there was mixed quality of methods and reporting. Approximately 31.4% (95% confidence interval [CI] 22.5–41.1; 6920 devices) of devices experienced a CVAD-associated complication, and 14.8% (95% CI 10.2–20.1; 24 studies; 11,762 devices) of CVADs failed before treatment completion (incidence rate (IR) of 0.5 per 1000 catheter days (95% CI 0.3–0.8; 12 studies; 798,000 catheter days)). Overall, 21.2% (95% CI 14.3–28.9; 26 studies; 5054 devices) of CVADs developed a CLABSI, with an IR of 0.9 per 1000 catheter days (95% CI 0.6–1.3; 12 studies; 798,094 catheter days). Tunneled central venous catheters (TCVC) and peripherally inserted central catheters (PICCs) were associated with increased complications in comparison to totally implanted venous access devices (TIVADs).

**Conclusion:**

CVAD complication rates in this population remain high. TCVCs and PICCs are associated with increased complications relative to TIVADs. Insufficient evidence exists to guide device selection in this cohort, necessitating further research to determine the role of PICCs in pediatric cancer care.

PROSPERO: CRD42022359467.

Date of registration: 22 September 2022.

**Supplementary Information:**

The online version contains supplementary material available at 10.1007/s00520-024-08853-0.

## Introduction

Globally, an estimated 400,000 children and adolescents (0–19 years) are diagnosed with cancer annually [1]. Central venous access devices (CVADs) are essential in facilitating anti-cancer and supportive therapies. CVAD type and timing of insertion vary greatly and are influenced by multiple factors including individual patient circumstances, cancer type, treatment required, proceduralist availability (i.e., surgeon, anesthetist, or nurse practitioner), and clinician preference. The chosen device may include a peripherally inserted central catheter (PICC), totally implantable venous access device (TIVAD), or tunneled central venous catheter (TCVC) [2].

General pediatric data indicates that one in four patients with CVADs will experience a significant complication or device failure before completing therapy [3, 4]. Post-insertion complications include central line–associated bloodstream infection (CLABSI), CVAD-associated venous thromboembolism (VTE), local infection, mechanical complications, and dehiscence [4, 5]. These complications require treatment, resulting in delays to anti-cancer therapy and thus increasing morbidity and mortality [3–7]. Oncology patients are a distinct cohort whose susceptibility to complications is unique secondary to the cancer itself and treatments administered. Understanding the incidence of CVAD-associated complications in this cohort is important to help guide decision-making on device selection and where further research is needed to reduce adverse events.

This systematic review aimed to determine the current evidence regarding the incidence of CVAD-associated complications in pediatric patients with cancer.

## Methods

This study used standard methods for systematic reviews and is reported in accordance with the “Preferred Reporting Items for Systematic Reviews and Meta-Analyses: the PRISMA statement” [8] and the Meta-Analyses of Observational Studies in Epidemiology (MOOSE) checklist [9]. This study was registered with PROSPERO on 22 September 2022 (CRD42022359467).

### Eligibility criteria

A systematic search (see Online Resource 1) was conducted to look for studies examining incidence, failure, and/or post-insertion complications of CVADs in pediatric oncology patients. Procedural/insertion complications were not included. Studies were eligible for inclusion if they met the following criteria: (1) cohort design (prospective or retrospective) or (2) control arm of randomized controlled trials (RCTs), (3) failure and/or complications of CVADs included as an outcome measure, (4) pediatric patients aged 0 to 18 years, (5) patients with an oncological diagnosis, and (6) CVAD inserted for any length of time during their treatment. Studies were excluded if they were not written in English and/or if they were published prior to 2012 to best reflect current practices.

### Outcome measures

The primary outcome was CVAD complications as a composite (overall complications). The secondary outcomes were CVAD complications post-successful CVAD insertion:

1. CVAD failure [4, 10]
2. CLABSI [11]
3. Local CVAD infection [12]
4. Occlusion [13]
5. CVAD-associated VTE [13]
6. Dislodgement or migration [13]
7. Breakage and/or rupture [14]
8. Dehiscence [15]

Full description of definitions is available in Online Resource 2.

### Search strategy and study selection

The US National Library of Medicine National Institutes of Health (PubMed), Embase, and Cumulative Index of Nursing and Allied Health Literature (CINAHL) were systematically searched. Medical subject headings and searches were developed in conjunction with a healthcare librarian (see Online Resource 1) and screened for inclusion independently by two authors using Covidence [16]. References of full-text articles were reviewed to identify additional studies. Disagreements were resolved through review by a third author.

### Data extraction and missing data

Data extraction was performed by one reviewer using a standardized data extraction form, checked by a second author. The data fields extracted included country, study design, population, diagnosis, catheter type, frequency of CVAD failure and/or complications, catheter days, and CVAD risk factors. Where data were missing, study authors were contacted.

### Statistical methods

The proportion and corresponding 95% confidence interval (CI) of the different complications (overall complications, failure, CLABSI, local infection, VTE, occlusion, dehiscence, dislodgement/migration, and breakage/rupture) were calculated using a random effects model to consider effects from small studies. Where studies reported catheter days, the incidence rate (IR) and corresponding 95% CI were calculated as events per 1000 catheter days. The point estimates (with 95% CI’s) from separate datasets were pooled using the DerSimonian-Laird random effects method [17], with the variances of the raw proportions stabilized using the Freeman-Turkey double arcsine transformation [18, 19]. The prediction interval is also reported to reflect the uncertainty expected in the summary effect if a new study is included in the meta-analysis [20]. Between-study variations were assessed using (1) the Chi-square (Chi^2^) test of heterogeneity to evaluate whether the variation between studies exceeded that expected by chance, whereby *p* ≤ 0.01 indicated the presence of heterogeneity, and (2) the Higgins *I*^2^ statistic, to estimate the percentage of the total variation in effect estimates across the studies attributable to heterogeneity rather than chance [21]. Publication bias was examined using Funnel plots and Egger’s test. These analyses were conducted using R (version 4.2.3) [22].

### Subgroup analyses

Subgroup analysis was performed on CVAD type where data were available. There was insufficient data to subgroup by cancer diagnosis (solid tumor versus hematological malignancy) as originally planned.

### Risk of *bias* assessment

The Mixed Methods Appraisal Tool (MMAT) [23] was used by two reviewers to assess the quality of evidence for the studies included. Disagreements in rating were discussed with a third reviewer.

## Results

### Systematic search results

**Fig. 1:**
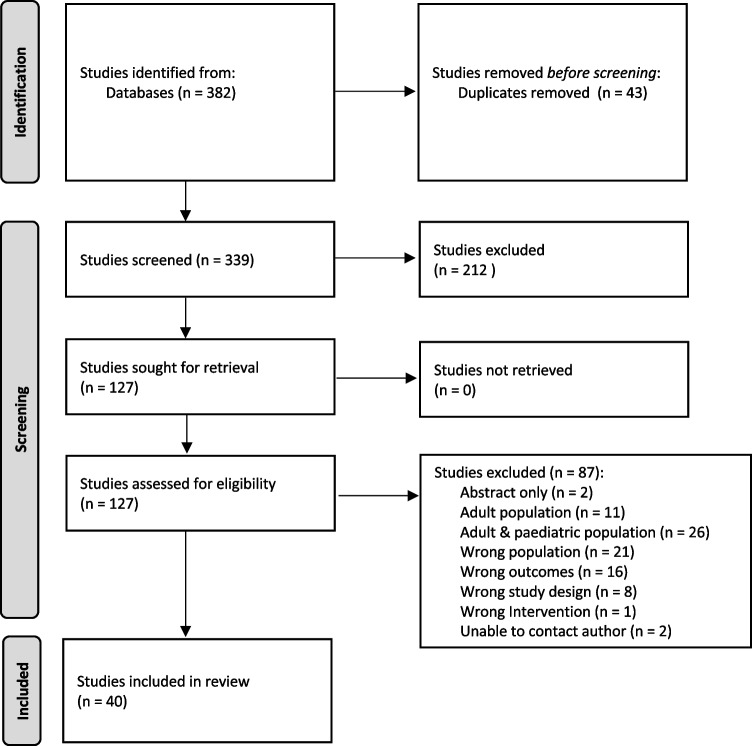
PRISMA flow diagram of study identification and selection

Figure 1 demonstrates the study selection process in accordance with the PRISMA guidelines [8]. A total of 382 studies were identified in the initial screening, and 40 studies met the inclusion criteria.

### Characteristics of included studies

Study characteristics are summarized in Table 1.. Of the 40 studies included, there were 29 (72.5%) retrospective cohort (RC) studies, 7 (17.5%) prospective cohort (PC) studies, 1 (2.5%) retrospective case–control (RCC) study, and 3 (7.5%) randomized control trials (RCT, control arm only). Fourteen (35%) studies included hematological malignancy only [24–36], 1 (2.5%) study included solid tumor malignancy only [37], and the remaining 25 (62.5%) studies included all malignancies [5, 24, 38–60]. Studies were conducted across Asia and Pacific [40, 43, 45, 48, 51], North America [28, 30, 34, 38, 47, 53, 58, 61, 62], South America [46], Africa [60], and Europe [5, 24–26, 29, 31–33, 36, 37, 39, 41, 42, 44, 49, 50, 52, 54–57, 59, 63].

**Table 1.** Study characteristics

Citation	Year	Country	Design	Population	Patients (*N*)	CVAD total (*N*)	CVAD type (*N*)	CVAD complications studied
Abate	2014	Italy	PC	Solid tumor	155	155	153 TCVC 2 TIVAD	1, 3, 4, 5, 6, 7
Albisetti	2013	Switzerland	PC	All malignancy	114	114	114 TIVAD	6
Beck	2019	Germany	RC	All malignancy	296	296	169 TCVC 127 TIVAD	1, 3, 4, 6, 7, 8
Berrueco	2013	Spain	PC	All malignancy	73	73	73 TIVAD	3, 4
Bratton	2014	USA	RC	All malignancy	170	178	34 TCVC 34 PICC 110 TIVAD	1, 2, 3, 7, 8
Buonpane	2022	USA	RC	All malignancy	6553	6553	3803 TCVC/ PICC 2750 TIVAD	1, 2, 3, 4
Celebi	2013	Turkey	RC	All malignancy	31	31	31 CVADs	2, 3, 4
Cesca	2014	Italy	RC	Hematological	117	117	117 CVADs	1, 2, 3, 4, 5, 6, 7, 8
Cher	2022	Singapore	RC	All malignancy	243	243	243 TIVAD	3
Fu	2016	USA	RC	Hematological	198	292	52 TCVC 240 TIVAD	1, 2, 3, 4, 6, 7
Gidl	2022	Austria	RCT	Hematological	1026	1026	1026 CVADs	6
Gonzalez	2012	USA	RC	Hematological	172	172	139 TCVC 33 TIVAD	1, 3, 4, 7
Gowin	2020	Poland	RC	All malignancy	241	277	277 TIVAD	2, 3
Jarvis	2019	Norway	PC	Hematological	47	47	47 CVADs	6
Khera	2022	India	PC	All malignancy	61	61	61 TCVC	1, 2, 3, 4, 7, 8
Kristinsdottir	2021	Iceland	RC	All malignancy	94	131	49 TCVC 82 TIVAD	1, 2, 3
Lücking	2013	Denmark	RC	Hematological	31	31	31 CVADs	3
Mangum	2013	USA	RC	All malignancy	743	878	475 TCVC 403 TIVAD	1, 2, 3
Martynov	2018	Germany	RC	All malignancy	238	273	273 TCVC	1, 5, 7
Martynov	2021	USA	RC	Hematological	350	498	498 TCVC	1, 3, 4, 7
Miliaraki	2017	Greece	RC	All malignancy	91	91	91 CVADs	3
Moell	2019	Sweden	RC	All malignancy	154	154	11 TCVC 143 TIVAD	3
Mokone	2021	South Africa	RC	All malignancy	293	293	293 TCVC	2, 3, 5, 8
Noailly Charny	2018	France	RC	Hematological	192	295	157 PICC 138 CVADs	6
Onyeama	2018	USA	RC	Hematological	198	438	228 PICC 210 CVADs	6
Park	2021	South Korea	RC	All malignancy	470	470	226 TCVC 242 TIVAD 2 NS	3
Redkar	2019	India	RC	All malignancy	69	72	72 TIVAD	1, 3, 5
Rogers	2017	USA	RCC	Hematological	40	40	16 TCVC 24 TIVAD	3
Ruiz-Llobet	2022	Spain	RC	Hematological	652	652	652 CVADs	6
Rykov	2018	Russia	PC	All malignancy	353	353	353 PICC	1, 2, 3, 5, 6, 7
Schoot	2016	Netherlands	RCT	All malignancy	305	305	18 TCVC 287 TIVAD	2, 3, 5
Schoot	2015	Netherlands	RCT	All malignancy	154	154	9 TCVC 145 TIVAD	3
Ullman	2020	Australia	PC	All malignancy	56	56	23 TCVC 16 PICC 17 TIVAD	5
Van Den Bosch	2019	Netherlands	RC	All malignancy	201	307	307 CVADs	1, 3, 4, 6, 7, 8
Van Den Bosch	2022	Netherlands	RC	Hematological	98	98	98 CVADs	1, 3, 4, 6, 7, 8
Viana Taveira	2017	Brazil	RC	All malignancy	188	224	224 TIVAD	3
White	2012	UK	RC	Hematological	322	322	68 TCVC 254 TIVAD	1, 2, 5, 7, 8, 9
Wiegering	2014	Germany	RC	All malignancy	269	269	269 CVADs	6
Zachariah	2014	Oman	RC	Hematological	29	42	3 TCVC 28 TIVAD 11 NS	1, 2, 3
Zakhour	2017	USA	RC	All malignancy	92	92	92 CVADs	3

Abbreviations:* CVAD* central venous access device, *PC* prospective cohort, *RC* retrospective cohort, *RCC* retrospective case–control, *RCT* randomized control trial, *USA* United States of America, *TCVC* tunneled central venous catheter, *TIVAD* totally implanted venous access devices, *PICC* peripherally inserted central catheters, *N* number, *NS* not specified, 1 Overall complications, 2 = Failure, 3 = CLABSI, 4 = Local infection, 5 = Occlusion, 6 = CVAD-associated VTE, 7 = Dislodgement/migration, 8 = Breakage and/or rupture, 9 = Dehiscence.

### Study quality

The MMAT tool [23] was used to assess the quality of the studies, and overall, the quality of the studies included was mixed, as summarized in Table 2. There were several studies that did not provide adequate definitions for outcomes. Redkar et al. [43] were contacted and able to provide a definition for CLABSI but not for other complications; therefore, only data for CLABSI were included in the analysis. Only device failure data was able to be included for both Buonpane et al. [53] and Mangum et al. [47] as other definitions were unable to be clarified by the authors. Three studies grouped CLABSI and local infection together, and as these were not able to be clarified, data from these outcomes were not included [24, 36, 38]. One study did not meet the criteria for CLABSI and was excluded [25]. Several studies were unable to clarify if patients with CVAD-associated VTE were symptomatic and thus were excluded [5, 34, 35, 38, 48].

**Table 2:** Study quality assessment

Citation	Year	S1. Are there clear research questions?	S2. Do the collected data allow to address the research questions?	3.1 Are the participants representative of the target population?	3.2 Are the measurements appropriate regarding both the outcome and intervention?	3.3 Are there complete outcome data?	3.4 Are the confounders accounted for in the design and analysis?	3.5 During the study period, is the intervention administered (or exposure occurred) as intended?
Abate	2014	Y	Y	Y	Y	Y	Y	Y
Albisetti	2013	Y	Y	Y	Y	Y	Y	Y
Beck	2019	Y	Y	Y	Y	Y	Y	Y
Berrueco	2013	Y	Y	Y*	Y	Y	Y	Y
Bratton	2014	Y	Y	Y	N	Y	Y	Y
Buonpane	2022	Y	Y	Y	N	Y	Y	Y
Celebi	2013	Y	Y	Y*	Y	Y	Y	Y
Cesca	2014	Y	Y	Y	Y	N	Y	Y
Cher	2022	Y	Y	Y	Y	Y	Y	Y
Fu	2016	Y	Y	Y	N	N	Y	Y
Gidl	2022	Y	Y	Y	N	Y	Y	Y
Gonzalez	2012	Y	Y	Y	Y	Y	Y	Y
Gowin	2020	Y	Y	Y	Y	Y	Y	Y
Jarvis	2019	Y	Y	Y*	Y	N	Y	Y
Khera	2022	Y	Y	Y*	Y	Y	Y	Y
Kristinsdottir	2021	Y	Y	Y*	Y	Y	Y	Y
Lücking	2013	Y	Y	Y*	N	Y	Y	Y
Mangum	2013	Y	Y	Y	N	Y	Y	Y
Martynov	2018	Y	Y	Y	N	Y	Y	Y
Martynov	2021	Y	Y	Y	Y	Y	Y	Y
Miliaraki	2017	Y	Y	Y*	Y	Y	Y	Y
Moell	2019	Y	Y	Y	Y	Y	Y	Y
Mokone	2021	Y	Y	Y	Y	Y	Y	Y
Noailly Charny	2018	Y	Y	Y	Y	Y	Y	Y
Onyeama	2018	Y	Y	Y	Y	Y	Y	Y
Park	2021	Y	Y	Y	Y	Y	Y	Y
Redkar	2019	Y	Y	Y*	N	Y	Y	Y
Rogers	2017	Y	Y	Y*	Y	Y	Y	Y
Ruiz-Llobet	2022	Y	Y	Y	Y	Y	Y	Y
Rykov	2018	Y	Y	Y	Y	Y	Y	Y
Schoot	2016	Y	Y	Y	Y	N	Y	Y
Schoot	2015	Y	Y	Y	Y	N	Y	Y
Ullman	2020	Y	Y	Y*	Y	Y	Y	Y
Van Den Bosch	2019	Y	Y	Y	Y	Y	Y	Y
Van Den Bosch	2022	Y	Y	Y	Y	Y	Y	Y
Viana Taveira	2017	Y	Y	Y	Y	Y	Y	Y
White	2012	Y	Y	Y	N	N	Y	Y
Wiegering	2014	Y	Y	Y	Y	Y	Y	Y
Zachariah	2014	Y	Y	Y*	Y	Y	Y	Y
Zakhour	2017	Y	Y	Y*	Y	Y	Y	Y

Abbreviations:* Y* Yes, *N* No.

*Population size < 100.

### Outcomes

Table 3 reports the pooled proportions and IRs of CVAD-associated complications.

**Table 3:** Subgroup analyses: proportion and incidence rates of CVAD-associated complications by device type

Event	Proportion of complications						Incidence rates of complications per 1000 catheter days					
Device	Studies	CVADs	Outcomes	Pooled %	95% CI	Prediction interval	Studies	Catheter days	Outcome	Pooled IR	95% CI	Prediction interval
Overall												
Total	33	6920	1992	31.4^a,f,h^	22.5–41.1	0.0–88.3	14	840,688	1476	2.3^a, f, g^	1.6–3.2	0.2–6.8
All	21	3858	1099	34.3^a,f^	20.1–50.1		9	434,900	932	2.9^a, f^	1.6–4.6	
TIVAD	7	1289	386	23.8^a,f^	8.2–44.3		3	267,898	298	1.3^a, e^	0.9–1.8	
TCVC	5	1092	342	33.1^a,f^	23.0–44.0		2	137,890	246	1.8^b, d^	1.5–2.1	
TCCVC	1	293	110	37.5	32.0–43.4		-	-	-	-	-	
PICC	2	388	55	22.7^a,f^	2.9–52.6		-	-	-	-	-	
Failure												
Total	24	11,762	2489	14.8^a,f,g^	10.2–20.1	0.00–48.8	12	798,000	399	0.5^a, f, g^	0.3–0.8	0.0–2.2
All	14	8605	2017	15.3^a,f^	9.7–21.8		9	437,684	305	0.7^a, f^	0.4–1.1	
TIVAD	6	1340	177	8.8^a,f^	1.5–20.7		2	260,635	90	0.2^a, f^	0.0–0.8	
TCVC	5	1136	171	21.2^a,f^	4.1–46.2		1	99,681	4	0.0	0.0–0.1	
TCCVC	1	293	89	30.4	25.2–36.0		-	-	-	-	-	
PICC	2	388	35	9.4^b,c^	4.6–15.6		-	-	-	-	-	
CLABSI												
Total	26	5052	989	21.2^a,f,g^	14.3–28.9	0.0–67.7	12	798,094	694	0.9^a, f, g^	0.6–1.3	0.0–2.7
All	17	2562	464	23.4^a,f^	14.4–33.7		8	430,515	307	0.7^a, f^	0.3–1.1	
TIVAD	5	1058	248	18.1^a,f^	5.2–36.3		3	267,898	222	1.2^a, f^	0.6–2.0	
TCVC	3	785	238	30.4^a,f^	21.3–40.3		1	99,681	165	1.7	1.4–1.9	
TCCVC	1	293	39	13.3	9.6–17.7		-	-	-	-	-	
PICC	1	354	0	0	0.0–1.0		-	-	-	-	-	
CVAD-associated VTE												
Total	12	4008	197	5.2^a,f,g^	2.2–9.3	0.0–26.5	3	147,455	9	0.0^b, c^	0.0–0.1	0.0–1.3
All	9	3376	120	3.0^a,f^	1.0–6.0		3	147,455	9	0.0^b, c^	0.0–0.1	
TIVAD	1	121	45	37.2	28.6–46.4		-	-	-	-	-	
PICC	2	511	32	6.9^a, f^	2.3–13.5		-	-	-	-	-	
Local infection												
Total	11	2497	114	3.9^a,f,h^	1.5–7.1	0.0–20.4	9	737,729	113	0.1^a, f, g^	0.0–0.3	0.0–0.8
All	7	1437	70	3.8^a,f^	0.8–8.5		6	377,413	71	0.2^a, f^	0.0–0.4	
TIVAD	2	501	38	7.3^b,d^	3.9–11.7		2	260,635	38	0.1^b, c^	0.1–0.2	
TCVC	2	559	6	1.3^b,d^	0.0–4.8		1	99,681	4	0.0	0.0–0.1	
Occlusion												
Total	11	2562	155	6.3^a,f,h^	3.8–9.3	0.0–20.3	7	513,716	113	0.8^a, f, g^	0.3–1.3	0.0–3.5
All	5	971	75	10.2^a,f^	3.4–20.0		5	292,413	87	1.4^a, f^	0.5–2.8	
TIVAD	3	603	18	2.7^b,c^	1.3–4.6		1	183,094	12	0.1	0.0–0.1	
TCVC	2	341	18	5.1^b,c^	2.9–7.8		1	38,209	14	0.4	0.2–0.6	
TCCVC	1	293	18	6.1	3.7–9.5		-	-	-	-	-	
PICC	1	354	26	7.3	4.9–10.6		-	-	-	-	-	
Dislodgement/migration												
Total	12	2785	176	4.6^a,f,h^	2.6–7.1	0.0–17.0	9	605,885	132	0.2^a, f, g^	0.1–0.4	0.0–1.1
All	6	1320	55	3.6^a,e^	2.1–5.5		7	384,582	58	0.1^a, f^	0.1–0.3	
TIVAD	3	641	36	3.1^a,f^	0.0–11.2		1	183,094	30	0.2	0.1–0.2	
TCVC	4	436	56	8.0^a,f^	2.2–16.4		1	38,209	44	1.2	0.8–1.5	
PICC	2	388	29	7.0^b,c^	4.5–9.9		-	-	-	-	-	
Breakage/rupture												
Total	9	2002	51	1.7^a,f,h^	0.5–3.5	0.0- 10.3	6	379,447	48	0.1^a, f^	0.0–0.3	0.0–1.2
All	5	1148	39	2.2^a,f^	0.3–5.7		6	379,447	48	0.1^a, f^	0.0–0.3	
TIVAD	2	364	2	0.4^b,c^	0.0–1.5		-	-	-	-	-	
TCVC	3	163	6	3.1^a,e^	0.0–11.1		-	-	-	-	-	
TCCVC	1	293	3	1.0	0.2–3.0		-	-	-	-	-	
PICC	1	34	1	2.9	0.1–15.3		-	-	-	-	-	
Dehiscence												
Total	1	322	17	2.4^a,f^	0.0–12.7	NA	-	-	-	-	-	
TIVAD	1	254	17	6.7	3.9–10.5		-	-	-	-	-	
TCVC	1	68	0	0.0	0.0–5.3		-	-	-	-	-	

Abbreviations: *NA* not applicable due to insufficient sample size.

Chi^2^ test of heterogeneity:^a^ = significant heterogeneity, ^b^ = nonsignificant heterogeneity.

Higgins I^2^ test of heterogeneity: ^c^ = nonsignificant heterogeneity (0–40%), ^d^ = moderate heterogeneity (30–60%), ^e^ = substantial heterogeneity (50–90%), ^f^ = considerable heterogeneity (75–100%).

Test for subgroup differences: ^g^ = significant heterogeneity, ^h^ = nonsignificant heterogeneity.

### Overall complications

Overall, 31.4% (95% CI 22.5–41.1; 33 studies, 6920 devices) of pediatric CVADs experienced a complication, with an IR of 2.3 per 1000 catheter days (95% CI 1.6–3.2; 14 studies; 840,688 catheter days; Table 3). There was a high degree of heterogeneity observed in the pooled data for both proportion (*I*^2^ = 99%; Chi^2^ = 2399, df = 32, *p* =  < 0.01, prediction interval [0.0–88.9]) and IR (*I*^2^ = 98%; Chi^2^ = 680.8, df = 13, *p* =  < 0.01, prediction interval [0.2–6.8]). Tunneled CVADs had the highest pooled proportion of overall complications (TCVC 33.1% [95% CI 23.0–44.0], 1092 CVADs; TCCVC 37.5% [95% CI 32.0–43.4]; 293 CVADs) and the highest pooled IR per 1000 catheter days (1.8 [95% CI 1.5–2.1], 137,890 catheter days). PICCs had the lowest pooled proportion of overall complications (22.7% [95% CI 2.9–52.6]; 358 CVADs), whereas TIVADs had the lowest pooled IR per 1000 catheter days (1.3 [95% CI 0.9–1.8]; 267,898 catheter days). Funnel plots for both proportions and rates were visually symmetrical (Egger’s test: *p* = 0.19 and 0.12, respectively).

### CVAD failure

Overall, 14.8% (95% CI 10.2–20.1; 24 studies; 11,762 devices; *I*^2^ = 98%; Chi^2^ = 943, df = 23, *p* =  < 0.01, prediction interval [0.0–48.8]) of CVADs failed prior to completion of planned therapy, with an IR of 0.5 per 1000 catheter days (95% CI 0.3–0.8; 12 studies; 798,000 catheter days (*I*^2^ = 96%; Chi^2^ = 290, df = 11, *p* =  < 0.01, prediction interval [0.0–2.2]; Table 3). Tunneled CVADs had the highest pooled proportion of device failure (TCVC 21.2% [95% CI 4.1–46.2], 1136 CVADs; TCCVC 30.4% [95% CI 25.2–36.0]; 293 CVADs). TIVADs had the highest pooled IR per 1000 catheter days of device failure (0.2 [95% CI 0.0–0.8]; 260,635 catheter days). Funnel plot for proportion was asymmetrical on visual inspection (*p* = 0.04), but symmetrical for rates (*p* = 0.59).

#### CLABSI

**Fig. 2:**
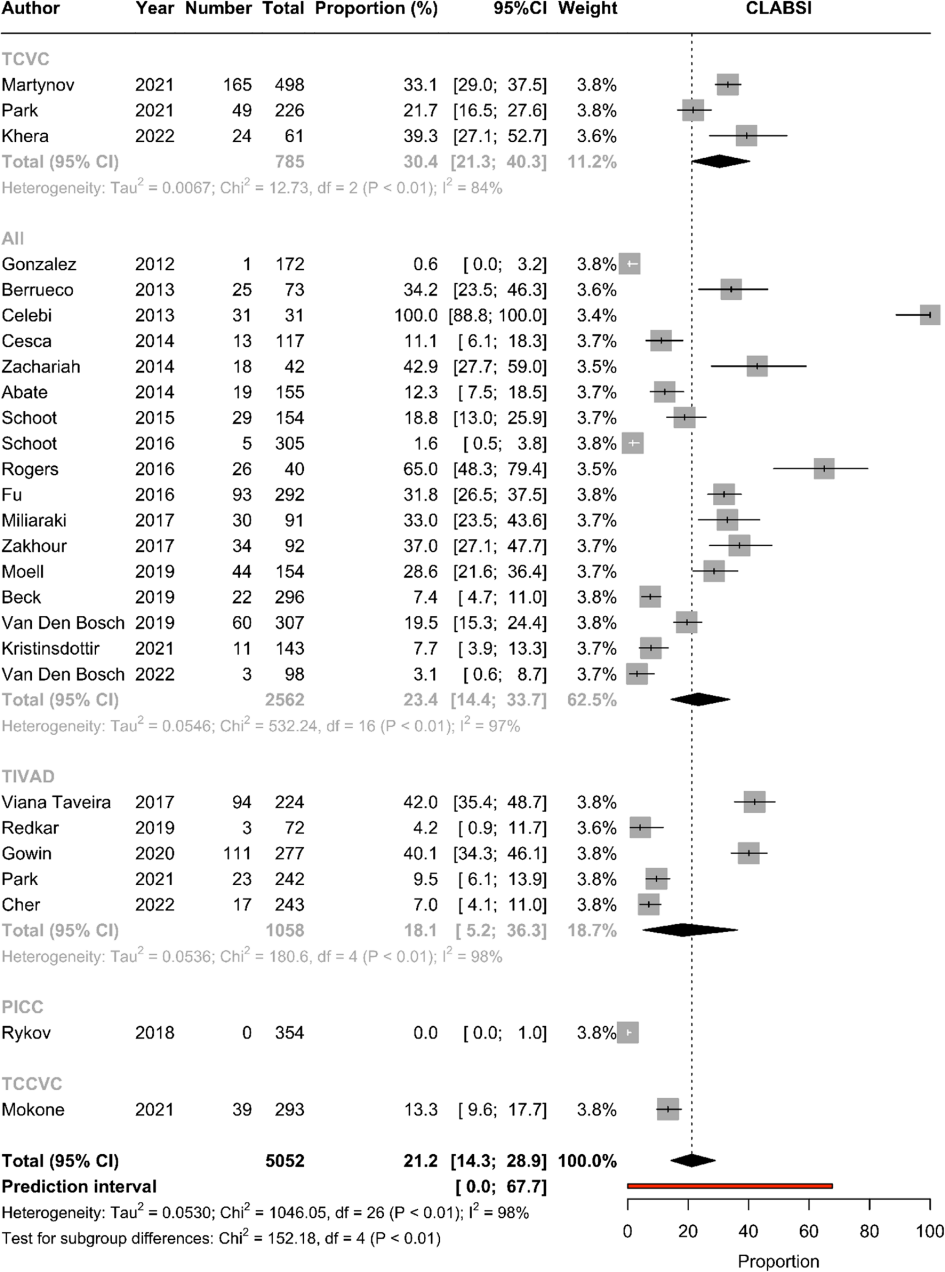
Pooled proportion of CLABSI by device subgroup. Abbreviations: CLABSI, central line–associated bloodstream infection; TCVC, tunneled central venous catheter; TIVAD, totally implanted venous access device; PICC, peripherally inserted central catheter; TCCVC, tunneled cuffed central venous catheter; CI, confidence interval

Overall, 21.2% (95% CI 14.3–28.9; 26 studies; 5052 devices; *I*^2^ = 98%; Chi^2^ = 1031, df = 25, *p* =  < 0.01, prediction interval [0.0–67.7]) of CVADs developed a CLABSI, with an IR of 0.9 per 1000 catheter days (95% CI 0.6–1.3; 12 studies; 798,094 catheter days; *I*^2^ = 96%; Chi^2^ = 267, df = 11, *p* =  < 0.01, prediction interval [0.0–2.7]; Table 3). Figure 2 demonstrates the pooled proportion by device type. TCVCs had the highest pooled proportion of CLABSI (30.4% [95% CI 21.3–40.3]; 785 CVADs) and the highest pooled IR per 1000 catheter days (1.7 [95% CI 1.4–1.9]; 99,681 catheter days). Funnel plot was symmetrical on visual inspection for both proportions and rates (*p* = 0.13 and 0.55, respectively).

### CVAD-associated VTE

Overall, 5.2% (95% CI 2.2–9.3; 12 studies; 4008 devices; *I*^2^ = 95%; Chi^2^ = 244, df = 11, *p* =  < 0.01, prediction interval [0.0–25.6]) of CVADs developed a VTE. The IR of VTE per 1000 catheter days was 0.0 (95% CI 0.0–0.1; 3 studies, 147,455 catheter days; *I*^2^ = 22%; Chi^2^ = 2.57, df = 2, *p* = 0.28, prediction interval [0.0–1.3]; Table 3). TIVADs had the highest pooled proportion of VTE (37.2% [95% CI 28.6–46.4]; 121 devices). No studies reported catheter days by device type; therefore, IRs for subgroup analysis could not be determined. Funnel plot was symmetrical on visual inspection for both proportions and rates (*p* = 0.14 and 0.14, respectively).

### Local infection

Overall, 3.9% (95% CI 1.5–7.1; 11 studies; 2497 devices; *I*^2^ = 92%; Chi^2^ = 120, df = 10, *p* =  < 0.01, prediction interval [0.0–20.4]) of CVADs experienced local infection, with an IR of 0.1 per 1000 catheter days (95% CI 0.0–0.3; 9 studies; 737,729 catheter days; *I*^2^ = 93%; Chi^2^ = 108, df = 8, *p* =  < 0.01; prediction interval [0.0–0.8]; Table 3). TIVADs had the highest pooled proportion of local infection (7.3% [95% CI 3.9–11.7); 501 CVADs) and the highest pooled IR per 1000 catheter days (0.1 [95% CI 0.1–0.2]; 260,635 catheter days). Funnel plot was symmetrical on visual inspection for both proportions and rates (*p* = 0.47 and 0.45, respectively).

### Occlusion

As shown in Table 3, overall, 6.3% (95% CI 3.8–9.3; 11 studies; 2562 devices; (*I*^2^ = 88%; Chi^2^ = 83.15, df = 10, *p* =  < 0.01; prediction interval [0.0–20.3]) of CVADs experienced occlusion. The IR of occlusion per 1000 catheter days was 0.8 (95% CI 0.3–1.3; 7 studies, 513,716 catheter days; (*I*^2^ = 97%; Chi^2^ = 202, df = 6, *p* =  < 0.01; prediction interval [0.0–3.5]). PICCs had the highest pooled proportion of occlusion (7.3% [95% CI 4.9–10.6]; 354 CVADs). TCVCs had the highest pooled IR per 1000 catheter days of occlusion (0.4 [95% CI 0.2–0.6]; 38,209 catheter days); although no studies reported catheter days for PICCs, therefore, IRs for PICCs could not be determined. Funnel plot was symmetrical on visual inspection for proportion (*p* = 0.16) but asymmetrical on visual inspection for rates (*p* < 0.01).

### Dislodgement/migration

Overall, 4.6% (95% CI 2.6–7.1; 12 studies; 2785 devices; *I*^2^ = 87%; Chi^2^ = 83, df = 11, *p* =  < 0.01; prediction interval [0.0–17.0]) of CVADs experienced dislodgement/migration, with an IR of 0.2 per 1000 catheter days (95% CI 0.1–0.4; 9 studies; 605,885 catheter days; *I*^2^ = 92%; Chi^2^ = 102, df = 12, *p* =  < 0.01; prediction interval [0.0–1.1]; Table 3). TCVCs had the highest pooled proportion of dislodgement/migration (8.0% [95% CI 2.2–16.4]; 436 CVADs) and the highest pooled IR per 1000 catheter days (1.2 [95% CI 0.8–1.5]; 38,209 catheter days). Funnel plot was symmetrical on visual inspection for both proportions and rates (*p* = 0.27 and 0.28, respectively).

### Breakage/rupture

As shown in Table 3, overall, 1.7% (95% CI 0.5–3.5; 9 studies; 2002 devices; *I*^2^ = 83%; Chi^2^ = 46.2, df = 8, *p* =  < 0.01; prediction interval [0.0–10.3]) of CVADs experienced breakage/rupture. The IR of breakage/rupture per 1000 catheter days was 0.1 (95% CI 0.0–0.3; 6 studies, 379,447 catheter days; *I*^2^ = 93%; Chi^2^ = 69, df = 5, *p* =  < 0.01; prediction interval [0.0–1.2]). TCVCs had the highest pooled proportion of breakage/rupture (3.1% [95% CI 0.0–11.1]; 163 CVADs). No studies reported catheter days by device type; therefore, IRs for device types could not be determined. Funnel plots were symmetrical on visual inspection for both proportions and rates (*p* = 0.93 and 0.43, respectively).

### Dehiscence

Dehiscence was only reported in one study [36]. The proportion of dehiscence in this study was 2.4% (95% CI 0.0–12.7; 1 study; 322 devices; Table 3). IR was unable to be calculated as catheter days were not reported. TIVADs had the highest proportion of dehiscence (6.7% [95% CI 3.9–10.5]; 254 CVADs).

## Discussion

This systematic review and meta-analysis is the first to provide a comprehensive overview of CVAD-associated complications in pediatric patients with cancer, incorporating data from 40 studies.

This meta-analysis demonstrated that overall, 31.4% (95% CI 22.5–41.1; 6920 devices) of CVADs experienced a complication, with 14.8% (95% CI 10.2–20.1; 24 studies; 11,762 devices) failing prior to treatment completion. Although no contemporary systematic reviews or meta-analysis data on CVAD complications in pediatric patients with cancer is available for comparison, these values are consistent with those reported in pediatric cohorts, where 20–30% of patients will experience a significant device complication [4]. Additionally, these results are also consistent with historical data from a 2005 prospective study by Fratino et al. [13] who reported 40% of pediatric patients with cancer experienced at least one complication of their CVAD. CVAD complications have significant burdens on the patient and healthcare system, leading to unplanned hospital admissions, additional treatments (e.g., antibiotics, anti-coagulants), and additional procedures (device replacement). Such interventions cause distress for caregivers and patients, costs to the healthcare system, and treatment delays and more recently have been linked with increased morbidity and mortality [3, 7]. A study by Athale et al. [7] found that after adjusting for age, sex, diagnostic era, and cancer type, CVAD dysfunction was an independent determinant of 5-year overall survival (OS) (HR 1.87; 95% CI 1.02–3.42, *p* = 0.043) and event-free survival (EFS) (HR1.96; 95% CI 1.23–3.41, *p* = 0.018) in pediatric patients with cancer. It is important to note that the “overall” complication rate in our study should be interpreted in the context of significant heterogeneity with study design, diagnosis, and complications reported. However, even with considering the study heterogeneity, the data shows that a large proportion of these patients are experiencing complications. Further prospective studies are needed to determine the etiology of CVAD dysfunction and interventions which can be implemented to reduce the morbidity and mortality associated with CVAD use in these patients.

Outside of “overall” complications, the largest amount of data in pediatric patients with cancer was in relation to CLABSI. Our data found CLABSI occurred at an IR of 0.9 per 1000 catheter days (95% CI 0.6–1.3). The CLABSI rate in the literature for pediatric oncology patients is reported as being 2.1 per 1000 catheter days [13]. There are several reasons this meta-analysis found a lower IR of CLABSI compared with previously reported data. Firstly, there have been significant changes in CVAD care practices that have been strongly driven to reduce infection associated with these devices [64]. Secondly, data in this meta-analysis is representative of “all malignancy,” and this number may change when separating hematological from solid tumor malignancy, which have historically had higher rates of infection secondary to their underlying disease process and associated treatments [5, 49, 52]. Subgroup analysis by malignancy type was unable to be conducted on this data secondary to insufficient studies reporting CVAD complications by malignancy type. Thirdly, as already highlighted, this meta-analysis had minimal data in relation to PICCs, including no catheter days relating to CLABSI for PICCs despite PICCs being an increasingly utilized device in pediatric cancer care. It is known that CVAD dysfunction, e.g., occlusion and VTE, is associated with increased rates of infection [7] and without having sufficient data on PICCs and their complications, our CLABSI rate may be underrepresented. CLABSI results in increased healthcare costs and burden on patients and families with prolonged hospitalization, antibiotic treatment, and potential removal and replacement of their device [13]. CLABSI remains a significant burden to this population, and as our CVAD type and use have expanded, we also need to expand our understanding of this complication across all devices to help guide decision-making on device selection and infection prevention.

This review and meta-analysis also sought to understand CVAD-associated complications specific to device subgroups. Overall, TCVCs had the highest proportion of overall complications (33.1%, 95% CI 23.0–44.0), device failure (TCCVCs 30.4%, 95% CI 25.2–36.0; TCVC 21.2%, 95% CI 4.1–46.2), CLABSI (30.4%, 95% CI 21.3–40.3), dislodgement/migration (8%, 95% CI 2.2–16.4), and breakage/rupture (3.1%, 95% CI 0.0–11.1). It appeared that TIVADs, with the exception of CVAD-associated VTE and local infection, were associated with the lowest proportion of complications. There was less published data on PICCs, but PICCs had the highest proportion of occlusion (7.3%, 95% CI 4.9–10.6). The data on IRs by device type are less reliable as only 15 studies (37.5%) reported catheter days, and thus, it is difficult to draw meaningful conclusions from these results. Consistent with our data, though not specific to the oncology population, a systematic review in pediatric patients by Ullman et al. [4] found that TCVCs and PICCs were associated with a higher proportion of overall complications for failure, CLABSI, occlusion, and dislodgement/migration relative to TIVADs. A recent study was published to provide guidance on appropriate device selection in pediatric patients titled The Michigan Appropriateness Guide for Intravenous Catheters in Pediatrics: miniMAGIC [2]. The panel recommended the use of either TCVCs or TIVADs (if > 10 kg) as appropriate in pediatric patients with cancer. The decision between a TIVAD and a TCVC for therapy is determined by the treating team and is based on the diagnosis and therapy required. Over time, PICCs have emerged as an additional device for the delivery of anti-cancer therapy in this cohort. The use of PICCs across all ages was rated by the miniMAGIC panel as uncertain due to concerns relating to procedural and post-insertion complications such as infection and thrombosis [2]. Whilst the panel deemed it appropriate to place a PICC to commence urgent therapy for cancer (not compatible with a peripheral device), the appropriateness of doing this routinely was uncertain [2]. The available data from this systematic review and meta-analysis highlights the limited studies available on PICCs in this cohort and the lack of evidence to guide device decision-making in this population. It is also evident from this review that there is huge clinical and statistical heterogeneity in this cohort, and careful decision-making is needed when selecting devices, which is currently being driven by health service availability and preference and not necessarily on evidence. Further prospective research is needed comparing the use of and complications of PICCs in comparison with TCVCs and TIVADs to determine their role in this cohort.

This review highlighted the lack of good quality evidence surrounding CVAD-associated complications in pediatric patients with cancer. This lack of evidence is twofold; firstly, there is limited data available looking at CVAD complications within this specific cohort. As a result, the data that is available is mostly retrospective and confounded by many factors, such as the inability to subgroup by diagnosis or treatment, the inability to subgroup by device type across all studies, and differences in CVAD care and maintenance. This will likely improve in time; there is a trend of increasing numbers of CVAD research in this cohort over time, with 65% of the included studies in this review being conducted within the last 5 years. Secondly, there are significant issues with the accurate collection and reporting of vascular access data in these patients. As highlighted throughout this review, several studies had to be excluded or only limited data included as a result of poor outcome definition and/or incomplete data collection. In addition, our definitions (see online resource 2 – outcome definitions) had to be kept broad to enable us to capture the current scope of literature in this field. In 2021, Schults et al. [65] proposed an international consensus on the minimum details of reporting on vascular access research, including patient demographics, device characteristics, insertion details, CVAD care, and complications. Future research in this field needs to hold researchers accountable to the minimum standard of vascular reporting to enable us to accurately understand the incidence of CVAD-associated complications and why they are occurring.

## Limitations

The results of this systematic review and meta-analysis should be interpreted in the context of its limitations. Firstly, only 37.5% of studies reported catheter days, which limited the number of studies that could be included in the meta-analysis. Secondly, there was significant heterogeneity in the study populations utilized. 62.5% of studies included “all” malignancies. Hematological and solid tumor malignancies in pediatrics have significant differences not only in the pathophysiology of the disease but also in the chemotherapy and treatment administered, making generalizability difficult. There is also inconsistency internationally around the standard of care for vascular device maintenance. Improved and accurate reporting will help to address this issue. Due to the small numbers of reporting, subgroup analyses were difficult, making attempts to reduce heterogeneity difficult. The heterogeneity of this cohort was also reflected in the level of statistical heterogeneity seen in the analyses. Thirdly, whilst an attempt to keep the data relevant to modern CVAD care was made by limiting studies to the last 10 years, practices for CVAD care have evolved, and there is a risk that the pooled data may not accurately represent current rates of complications. We also elected to only include studies published in English, and there is a risk that as a result some data in this field may be missed. Finally, whilst our study reports associations between CVAD devices and complications, these do not reflect causation.

## Conclusions

The results from this systematic review and meta-analysis highlight the paucity of good quality evidence on CVAD-associated complications in pediatric patients with cancer. What is clear from the results available is that complication rates in this population remain high. Further prospective cohort studies assessing rates of complications within specific pediatric oncology cohorts and by device type are needed so that interventions can be implemented to reduce the morbidity and mortality associated with CVAD dysfunction. Future studies involving RCTs focused on device selection in different pediatric cancer cohorts would be beneficial to enable evidence-based device selection in these patients, particularly to determine the role of PICCs in pediatric cancer care. In addition, we need to hold researchers accountable to the minimum standards of reporting for vascular access studies, in order to improve the quality of research.

## Supplementary Information

Below is the link to the electronic supplementary material.

Supplementary file1 (DOCX 22 KB)

Supplementary file2 (DOCX 16 KB)

## Data Availability

Data are available from the corresponding author upon reasonable request.

## Abbreviations

CVAD: Central venous access devices
CLABSI: Central line–associated bloodstream infection
VTE: Venous thromboembolism
TCVC: Tunneled central venous catheters
PICC: Peripherally inserted central catheters
TIVAD: Totally implanted venous access devices
RCT: Randomized control trial
CI: Confidence interval
IR: Incidence rate
RC: Retrospective cohort
PC: Prospective cohort
RCC: Retrospective case–control
