# Supplementary material for: Central venous access device adverse events in pediatric patients with cancer: a systematic review and meta-analysis
Source: Support Care Cancer. 2024 Sep 16;32(10):662. doi: 10.1007/s00520-024-08853-0 (PMC11405478; doi:10.1007/s00520-024-08853-0)
Supplement: Supplementary file 1 — Supplementary file1 (DOCX 22 KB) [file 520_2024_8853_MOESM1_ESM.docx]

**Central Venous Access Device Adverse Events in Pediatric Patients with Cancer: A Systematic Review and Meta-Analysis**

Supportive Care in Cancer

**Authors:**

Jenna Nunn^1, 2, 3^

Mari D Takashima ^1,2^

Erin Wray-Jones ^4^

Trisha Soosay Raj ^1,2^

Diane M T Hanna ^5, 6, 7^

Amanda J Ullman ^1,2^

**Affiliations:**

^1^ Children’s Health Queensland Hospital & Health Service, Brisbane, Australia

^2^ The University of Queensland, Brisbane, Australia

^3^ Griffith University, Gold Coast, Australia

^4^ Sunshine Coast University Hospital

^5^ The University of Melbourne

^6^ Murdoch Children’s Research Institute

^7^ The Walter & Eliza Hall Institute

**Corresponding Author:**

Jenna Nunn

ORCID ID: 0000-0002-7790-9955

Queensland Children’s Hospital

501 Stanley Street, South Brisbane, Queensland, Australia, 4101

[Jenna.nunn2@health.qld.gov.au](mailto:Jenna.nunn2@health.qld.gov.au)

**Online Resource 1 – Systematic Search**

PUBMED Search

**Central venous line**

"Catheterization, Central Venous"[Mesh] OR "Central Venous Catheters"[Mesh] OR “Catheterization, Central Venous”[Mesh] OR “Central Catheterization”[tiab] OR “Central Catheterizations”[tiab] OR “Central Venous Catheterization”[tiab] OR “Central Venous Catheterizations”[tiab] OR CVC[tiab] OR CVL[tiab] OR CVCs[tiab] OR “Central Vein Catheterization”[tiab] OR “Central Vein Catheterizations”[tiab]

**Adverse event**

"adverse effects"[MeSH Subheading] OR adverse effect* OR adverse event* OR complication*[tiab]) OR (((((("Bacteremia"[Mesh]) OR "Infections"[Mesh]) OR "Thromboembolism"[Mesh]) OR "Surgical Wound Dehiscence"[Mesh]) OR "Device Removal"[Mesh]) OR "Catheter-Related Infections"[Mesh])

**Risk**

Risk*[tiab] OR risk[mh] OR risk factors[mh]

**Pediatric**

"Infant"[Mesh] OR "Child"[Mesh] OR "Adolescent"[Mesh] OR "Child, Preschool"[Mesh] OR "Infant, Newborn"[Mesh] OR Infan*[tiab] OR newborn*[tiab] OR new-born*[tiab] OR perinat*[tiab] OR neonat*[tiab] OR baby[tiab] OR baby*[tiab] OR babies[tiab] OR toddler*[tiab] OR child[tiab] OR child*[tiab] OR children*[tiab] OR adolescen*[tiab] OR juvenil*[tiab] OR youth*[tiab] OR teen*[tiab] OR pubescen*[tiab] OR pediatrics[mh] OR pediatric*[tiab] OR paediatric*[tiab] OR prematur*[tiab] OR preterm*[tiab]

**Oncology**

"Neoplasms"[Mesh] OR tumor*[tiab] OR Tumour*[tiab] "Leukemia"[Mesh] OR "Lymphoma"[Mesh] OR "Wilms Tumor"[Mesh] OR "Neuroblastoma"[Mesh] OR "Rhabdomyosarcoma"[Mesh] OR "Hepatoblastoma"[Mesh] OR "Medulloblastoma"[Mesh] OR "Retinoblastoma"[Mesh] OR "Meningioma"[Mesh] OR "Glioma"[Mesh] OR "Sarcoma"[Mesh] OR "Central Nervous System Neoplasms"[Mesh] OR "Brain Neoplasms"[Mesh]

**Final Pubmed Search:**

(((("Catheterization, Central Venous"[Mesh] OR "Central Venous Catheters"[Mesh] OR "Catheterization, Central Venous"[Mesh] OR "Central Catheterization"[tiab] OR "Central Catheterizations"[tiab] OR "Central Venous Catheterization"[tiab] OR "Central Venous Catheterizations"[tiab] OR CVC[tiab] OR CVL[tiab] OR CVCs[tiab] OR "Central Vein Catheterization"[tiab] OR "Central Vein Catheterizations"[tiab]) AND (("adverse effects"[MeSH Subheading] OR adverse effect* OR adverse event* OR complication*[tiab]) OR (((((("Bacteremia"[Mesh]) OR "Infections"[Mesh]) OR "Thromboembolism"[Mesh]) OR "Surgical Wound Dehiscence"[Mesh]) OR "Device Removal"[Mesh]) OR "Catheter-Related Infections"[Mesh]))) AND (Risk*[tiab] OR risk[mh] OR risk factors[mh])) AND ("Infant"[Mesh] OR "Child"[Mesh] OR "Adolescent"[Mesh] OR "Child, Preschool"[Mesh] OR "Infant, Newborn"[Mesh] OR Infan*[tiab] OR newborn*[tiab] OR new-born*[tiab] OR perinat*[tiab] OR neonat*[tiab] OR baby[tiab] OR baby*[tiab] OR babies[tiab] OR toddler*[tiab] OR child[tiab] OR child*[tiab] OR children*[tiab] OR adolescen*[tiab] OR juvenil*[tiab] OR youth*[tiab] OR teen*[tiab] OR pubescen*[tiab] OR pediatrics[mh] OR pediatric*[tiab] OR paediatric*[tiab] OR prematur*[tiab] OR preterm*[tiab])) AND ("Neoplasms"[Mesh] OR tumor*[tiab] OR Tumour*[tiab] "Leukemia"[Mesh] OR "Lymphoma"[Mesh] OR "Wilms Tumor"[Mesh] OR "Neuroblastoma"[Mesh] OR "Rhabdomyosarcoma"[Mesh] OR "Hepatoblastoma"[Mesh] OR "Medulloblastoma"[Mesh] OR "Retinoblastoma"[Mesh] OR "Meningioma"[Mesh] OR "Glioma"[Mesh] OR "Sarcoma"[Mesh] OR "Central Nervous System Neoplasms"[Mesh] OR "Brain Neoplasms"[Mesh])

Embase Search

**Central venous line**

'central venous catheterization'/exp OR 'central venous catheter'/exp OR 'central venous catheter':ti,ab,kw OR 'catheterization':ti,ab,kw OR 'central venous catheterization':ti,ab,kw

**Adverse event**

'bacteremia'/exp OR OR 'wound dehiscence'/exp OR 'catheter infection'/exp OR 'device removal'/exp OR 'adverse event'/exp OR 'adverse event':ti,ab,kw OR 'complication':ti,ab,kw OR 'bacteremia':ti,ab,kw OR 'infection':ti,ab,kw OR 'venous thromboembolism':ti,ab,kw OR 'wound dehiscence':ti,ab,kw OR 'device removal':ti,ab,kw OR 'catheter infection':ti,ab,kw

**Risk**

'risk factor'/exp OR 'risk'/exp OR 'risk factor':ti,ab,kw OR 'risk':ti,ab,kw

**Pediatric**

'child'/exp OR 'adolescent'/exp OR 'child':ti,ab,kw OR 'adolescent':ti,ab,kw OR 'infant':ti,ab,kw OR 'newborn':ti,ab,kw OR 'baby':ti,ab,kw OR 'toddler':ti,ab,kw OR 'juvenile':ti,ab,kw OR 'pediatric':ti,ab,kw OR 'paediatric':ti,ab,kw

**Oncology**

'malignant neoplasm'/exp OR 'neoplasm':ti,ab,kw OR 'leukemia':ti,ab,kw OR 'lymphoma':ti,ab,kw OR 'nephroblastoma':ti,ab,kw OR 'neuroblastoma':ti,ab,kw OR 'rhabdomyosarcoma':ti,ab,kw OR 'hepatoblastoma':ti,ab,kw OR 'medulloblastoma':ti,ab,kw OR 'retinoblastoma':ti,ab,kw OR 'meningioma':ti,ab,kw OR 'glioma':ti,ab,kw OR 'sarcoma':ti,ab,kw OR 'central nervous system tumor':ti,ab,kw OR 'brain tumor':ti,ab,kw

**Final Embase search**

('central venous catheterization'/exp OR 'central venous catheter'/exp OR 'central venous catheter':ti,ab,kw OR 'catheterization':ti,ab,kw OR 'central venous catheterization':ti,ab,kw) AND ('bacteremia'/exp OR 'wound dehiscence'/exp OR 'catheter infection'/exp OR 'device removal'/exp OR 'adverse event'/exp OR 'adverse event':ti,ab,kw OR 'complication':ti,ab,kw OR 'bacteremia':ti,ab,kw OR 'infection':ti,ab,kw OR 'venous thromboembolism':ti,ab,kw OR 'wound dehiscence':ti,ab,kw OR 'device removal':ti,ab,kw OR 'catheter infection':ti,ab,kw) AND ('risk factor'/exp OR 'risk'/exp OR 'risk factor':ti,ab,kw OR 'risk':ti,ab,kw) AND ('child'/exp OR 'adolescent'/exp OR 'child':ti,ab,kw OR 'adolescent':ti,ab,kw OR 'infant':ti,ab,kw OR 'newborn':ti,ab,kw OR 'baby':ti,ab,kw OR 'toddler':ti,ab,kw OR 'juvenile':ti,ab,kw OR 'pediatric':ti,ab,kw OR 'paediatric':ti,ab,kw) AND ('malignant neoplasm'/exp OR 'neoplasm':ti,ab,kw OR 'leukemia':ti,ab,kw OR 'lymphoma':ti,ab,kw OR 'nephroblastoma':ti,ab,kw OR 'neuroblastoma':ti,ab,kw OR 'rhabdomyosarcoma':ti,ab,kw OR 'hepatoblastoma':ti,ab,kw OR 'medulloblastoma':ti,ab,kw OR 'retinoblastoma':ti,ab,kw OR 'meningioma':ti,ab,kw OR 'glioma':ti,ab,kw OR 'sarcoma':ti,ab,kw OR 'central nervous system tumor':ti,ab,kw OR 'brain tumor':ti,ab,kw) AND [english]/lim AND [embase]/lim AND ([article]/lim OR [article in press]/lim OR [review]/lim)

CINAHL search

**Central venous line**
MM "Central Venous Catheters"+ OR MM "Vascular Access Devices"  OR “Central Catheterization” OR “Central Catheterizations” OR “Central Venous Catheterization” OR “Central Venous Catheterizations” OR CVC OR CVL OR CVCs OR “Central Vein Catheterization” OR “Central Vein Catheterizations”

**Adverse event**
MH "Postoperative Complications+" OR MH "Adverse Health Care Event+" OR MM "Bacteremia" OR MH "Infection+" OR MH "Thromboembolism+" OR MM "Surgical Wound Dehiscence" OR MH "Device Removal+" OR MH "Catheter-Related Infections+" OR adverse effect* OR adverse event* OR complication

**Pediatric**
MH "Pediatric Care+" OR MH "Child+" OR MH "Adolescence+" OR MH "Infant+" OR MH "Pediatrics+" OR Infan* OR newborn* OR new-born* OR perinat* OR neonat* OR baby OR baby* OR babies OR toddler* OR child OR child* OR children* OR adolescen* OR juvenil* OR youth* OR teen* OR pubescen* OR pediatric* OR paediatric* OR prematur* OR preterm*

**Oncology**
MH "Neoplasms+" OR MH "Leukemia+" OR MH "Lymphoma+" OR MH "Wilms' Tumor+" OR MH "Neuroblastoma+" OR MH "Sarcoma+" OR MH "Brain Neoplasms+" OR MH "Central Nervous System Neoplasms+" OR tumor* OR Tumor*

**Final CINAHL search**
(MM "Central Venous Catheters"+ OR MM "Vascular Access Devices"  OR “Central Catheterization” OR “Central Catheterizations” OR “Central Venous Catheterization” OR “Central Venous Catheterizations” OR CVC OR CVL OR CVCs OR “Central Vein Catheterization” OR “Central Vein Catheterizations”) AND (MH "Postoperative Complications+" OR MH "Adverse Health Care Event+" OR MM "Bacteremia" OR MH "Infection+" OR MH "Thromboembolism+" OR MM "Surgical Wound Dehiscence" OR MH "Device Removal+" OR MH "Catheter-Related Infections+" OR adverse effect* OR adverse event* OR complication) AND (MH "Pediatric Care+" OR MH "Child+" OR MH "Adolescence+" OR MH "Infant+" OR MH "Pediatrics+" OR Infan* OR newborn* OR new-born* OR perinat* OR neonat* OR baby OR baby* OR babies OR toddler* OR child OR child* OR children* OR adolescen* OR juvenil* OR youth* OR teen* OR pubescen* OR pediatric* OR paediatric* OR prematur* OR preterm*) AND (MH "Neoplasms+" OR MH "Leukemia+" OR MH "Lymphoma+" OR MH "Wilms' Tumor+" OR MH "Neuroblastoma+" OR MH "Sarcoma+" OR MH "Brain Neoplasms+" OR MH "Central Nervous System Neoplasms+" OR tumor* OR Tumor*)
