# Supplementary material for: Central venous access device adverse events in pediatric patients with cancer: a systematic review and meta-analysis
Source: Support Care Cancer. 2024 Sep 16;32(10):662. doi: 10.1007/s00520-024-08853-0 (PMC11405478; doi:10.1007/s00520-024-08853-0)
Supplement: Supplementary file 2 — Supplementary file2 (DOCX 16 KB) [file 520_2024_8853_MOESM2_ESM.docx]

**Central Venous Access Device Adverse Events in Pediatric Patients with Cancer: A Systematic Review and Meta-Analysis**

Supportive Care in Cancer

**Authors:**

Jenna Nunn^1, 2, 3^

Mari D Takashima ^1,2^

Erin Wray-Jones ^4^

Trisha Soosay Raj ^1,2^

Diane M T Hanna ^5, 6, 7^

Amanda J Ullman ^1,2^

**Affiliations:**

^1^ Children’s Health Queensland Hospital & Health Service, Brisbane, Australia

^2^ The University of Queensland, Brisbane, Australia

^3^ Griffith University, Gold Coast, Australia

^4^ Sunshine Coast University Hospital

^5^ The University of Melbourne

^6^ Murdoch Children’s Research Institute

^7^ The Walter & Eliza Hall Institute

**Corresponding Author:**

Jenna Nunn

ORCID ID: 0000-0002-7790-9955

Queensland Children’s Hospital

501 Stanley Street, South Brisbane, Queensland, Australia, 4101

[Jenna.nunn2@health.qld.gov.au](mailto:Jenna.nunn2@health.qld.gov.au)

**Online Resource 2 – Outcome Definitions**

| Overall | The cumulative total number of complications (defined above) within the study cohort |
| --- | --- |
| CVAD failure | Failure of the device prior to completion of planned therapy [5, 11] |
| Central Line Associated Blood Steam Infection (CLABSI) | A laboratory confirmed bloodstream infection that is not secondary to an infection at another body site [12] |
| Local CVAD infection | Presence of erythema, swelling, tenderness at the insertion site +/- fever [12] |
| Occlusion | Either complete occlusion (unable to aspirate AND inject) or partial occlusion (unable to aspirate OR inject) [13] |
| CVAD-associated venous thromboembolism (VTE) | ultrasound evidence and clinical symptoms (pain/erythema/swelling/line dysfunction) of CVAD-associated VTE [13] |
| Dislodgement or migration | Any movement resulting in the CVAD migrating out of a central vein (central veins include lower 1/3 superior vena cava (SVC), right atrium (RA), SVC/RA junction or inferior vena cava (IVC) for lower limb insertion) [13] |
| Breakage and/or rupture | visible split/ break in the CVAD material resulting in external leakage and/or radiographic evidence of internal leak [14] |
| Dehiscence | Separation of the margins of surgical incision with/without protrusion of device [15] |

Abbreviations: CLABSI: central line-associated blood stream infection; CVAD: Central Venous Access Devices; IVC: inferior vena cava; RA: right atrium; SVC: superior vena cava; VTE: venous thromboembolism.
